# Adenovirus-Mediated Inducible Expression of a PD-L1 Blocking Antibody in Combination with Macrophage Depletion Improves Survival in a Mouse Model of Peritoneal Carcinomatosis

**DOI:** 10.3390/ijms22084176

**Published:** 2021-04-17

**Authors:** Maria Buñuales, Maria Cristina Ballesteros-Briones, Manuela Gonzalez-Aparicio, Sandra Hervas-Stubbs, Eva Martisova, Uxua Mancheño, Ana Ricobaraza, Sara Lumbreras, Cristian Smerdou, Ruben Hernandez-Alcoceba

**Affiliations:** 1 Program of Gene Therapy and Regulation of Gene Expression, Cima Universidad of Navarra, 31008 Pamplona, Spain; mbunara@unav.es (M.B.); mcbbriones@gmail.com (M.C.B.-B.); gamanuela@unav.es (M.G.-A.); emartisova@unav.edu (E.M.); aricobaraza@unav.es (A.R.); slumbreras@alumni.unav.es (S.L.); 2 Instituto de Investigacion Sanitaria de Navarra (IdiSNA), 31008 Pamplona, Spain; mshervas@unav.es (S.H.-S.); umancheno@unav.es (U.M.); 3 Program of Immunology and Immunotherapy, Cima Universidad de Navarra, 31008 Pamplona, Spain; 4 CIBERehd, Instituto de Salud Carlos III, Madrid, Spain

**Keywords:** PD-L1, immune checkpoint inhibitor, adenovirus, high-capacity adenoviral vector, colorectal cancer, peritoneal carcinomatosis, macrophages, clodronate, drug-inducible system, mifepristone

## Abstract

Immune checkpoint inhibitors (ICIs) have demonstrated remarkable efficacy in a growing number of malignancies. However, overcoming primary or secondary resistances is difficult due to pharmacokinetics issues and side effects associated with high systemic exposure. Local or regional expression of monoclonal antibodies (mAbs) using gene therapy vectors can alleviate this problem. In this work, we describe a high-capacity adenoviral vector (HCA-EFZP-aPDL1) equipped with a mifepristone-inducible system for the controlled expression of an anti-programmed death ligand 1 (PD-L1) blocking antibody. The vector was tested in an immune-competent mouse model of colorectal cancer based on implantation of MC38 cells. A single local administration of HCA-EFZP-aPDL1 in subcutaneous lesions led to a significant reduction in tumor growth with minimal release of the antibody in the circulation. When the vector was tested in a more stringent setting (rapidly progressing peritoneal carcinomatosis), the antitumor effect was marginal even in combination with other immune-stimulatory agents such as polyinosinic-polycytidylic acid (pI:C), blocking mAbs for T cell immunoglobulin, mucin-domain containing-3 (TIM-3) or agonistic mAbs for 4-1BB (CD137). In contrast, macrophage depletion by clodronate liposomes enhanced the efficacy of HCA-EFZP-aPDL1. These results highlight the importance of addressing macrophage-associated immunoregulatory mechanisms to overcome resistance to ICIs in the context of colorectal cancer.

## 1. Introduction

The programmed death 1 receptor (PD-1) is expressed in the surface of T cells in response to antigen stimulation, with the primary function of controlling the extent of activation to avoid excessive tissue damage [1]. PD-1 ligand 1 (PD-L1) is mainly expressed in antigen-presenting cells (APCs) and parenchymal cells of different organs, especially in the context of inflammation [2]. Binding of PD-L1 to PD-1 triggers phosphorylation of its immunoreceptor tyrosine-based inhibitory and switch motifs (ITIM and ITSM, respectively). This modification allows recruitment of Src homology phosphatase 2 (SHP-2) and reversion of the T cell activation program (reviewed in [3]). This inhibitory mechanism is usually exploited by the tumor microenvironment (TME) in order to resist the immune attack, usually by over-expression of PD-L1 [4]. Monoclonal antibodies (mAbs) with the ability to block the PD-1/PD-L1 axis, either binding to PD-1 (nivolumab, pembrolizumab) or PD-L1 (atezolizumab, durvalumab), are able to reverse this immunosuppressive state and obtain efficient anti-cancer immune responses (reviewed in [5]). These immune checkpoint inhibitors (ICIs) have improved the management of patients suffering from advanced melanoma [6], renal and urothelial cancers [7], non-small cell lung cancer [8], head and neck squamous cell carcinoma [9], Merkel cell carcinoma [10] and Hodgkin’s lymphoma [11]. However, less than 40% of patients obtain a clinical benefit from these treatments, due to primary or secondary resistances [3,12]. This is the case of colorectal cancer, in which only a minority of cases with high microsatellite instability and/or a high immunoscore are clear candidates for ICIs [13,14]. Dose escalation and combination with other ICIs or agonistic mAbs are limited by side effects, in part associated with the systemic exposure to these agents. One possibility to increase the efficacy and safety of ICIs is to use gene therapy vectors encoding the mAbs instead of the recombinant protein. Local or regional administration of these vectors can sustain expression of these therapeutic proteins, improving their pharmacokinetics. Recent examples of this approach include the use of adeno-associated (AAV) and Semliki Forest virus (SFV)-derived vectors expressing a blocking mAb against mouse PD-L1 (aPD-L1). An efficient antitumor effect was observed with SFV-aPDL1 in a syngeneic colorectal cancer model established by subcutaneous injection of MC38 cells [15]. These antecedents demonstrate the biological effect of the transgene and the sensitivity of the model towards PD-L1 blockade.

Current advances in surgery and interventional radiology allow access to tumors in virtually all anatomical locations [16]. However, the clinical feasibility of repeated administrations in internal organs is questionable. Therefore, the ability to sustain transgene expression for long periods of time is an important feature of vectors intended for ICI delivery. We have previously described the development of high-capacity adenoviral vectors (HC-AdV) equipped with a mifepristone-inducible system for the controlled expression of therapeutic genes. Using these tools, we could safely express strong immune-stimulatory genes such as interleukin 12 (IL-12) in mouse colorectal [17] and pancreatic cancer models [18,19]. 

In this work, we describe the development and characterization of an HC-AdV expressing aPD-L1 under the control of a recently described ubiquitous, fully humanized inducible system [19] (HCA-EFZP-aPDL1). We provide evidence that an efficient antitumor effect can be obtained in the subcutaneous MC38 model by controlled expression of aPD-L1 from the HCA-EFZP-aPDL1 vector. In order to investigate alternatives against anti-PD-L1 resistance, we employed a peritoneal carcinomatosis model in which the microenvironment and the high tumor burden reduce the efficacy of HCA-EFZP-aPDL1. We found that depletion of macrophages by clodronate liposomes obtains better results than combination with other ICIs such as anti-TIM3 or anti-CD137. 

## 2. Results

### 2.1. Controlled Expression of aPD-L1 Using an HC-AdV Vector Inhibits the Growth of Subcutaneous Tumors

**Figure 1:**
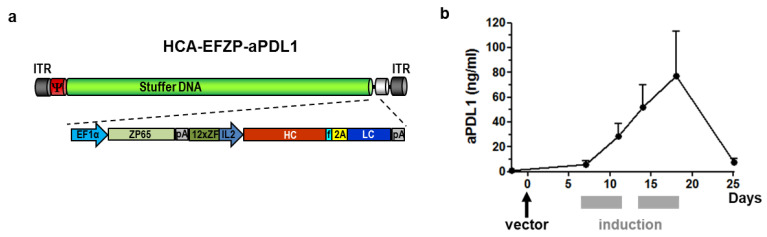
Characterization of the HCA-EFZP-aPDL1 vector. (**a**) Schematic representation of the vector genome. The vector backbone includes the inverted terminal repeats of adenovirus (ITR), the packaging signal (ψ) and stuffer DNA from human origin. The expression cassette comprises the inducible system for expression of the aPD-L1 coding sequence. 12xZF-IL2 is the inducible promoter based on a concatemer of ZF binding sites and the minimal interleukin-2 promoter. HC and LC are the heavy and light chains of IgG2a, linked by the 2A sequence from the foot and mouse disease virus and the furin recognition sequence (f). The ZP65 transactivator is expressed under the control of the elongation factor 1α promoter. pA, poly-adenylation signal. (**b**) The vector was administered by i.v. injection in C57BL/6 mice (1 × 10^10^ vg in 100 µL, *n* = 3), and one week later, the expression of aPD-L1 was activated by i.p. injection of mifepristone on days 7–11 and 14–18 (gray rectangles). The concentration of aPD-L1 in serum was quantified by ELISA and expressed as average ng/mL ± SEM.

The sequences coding for the heavy and light chains of a mAb against mouse PD-L1 (IgG2a) were linked using a 2A autoprotease sequence, as described previously [15]. This transgene was placed under the control of a fully humanized mifepristone-induced system designed to be functional in different tissues [19]. The expression cassette was inserted in an HC-AdV vector (HCA-EFZP-aPDL1), depicted in Figure 1a. For rapid evaluation of the vector functionality, C57BL/6 mice were injected intravenously (i.v.) with a low vector dose corresponding to 1 × 10^10^ viral genomes (vg), in 100 µL, and one week later, the expression of aPD-L1 was activated by intraperitoneal (i.p.) injection of mifepristone (RU486). The induction regime consisted of two cycles of five daily injections separated by 2 days. As shown in Figure 1b, the concentration of the aPD-L1 antibody in serum experienced a progressive increase from the initiation of induction until the last day of mifepristone administration, reaching a peak of 75 ng/mL. One week after the cessation of induction, the concentration of aPD-L1 dropped to basal levels (5 ng/mL). 

**Figure 2:**
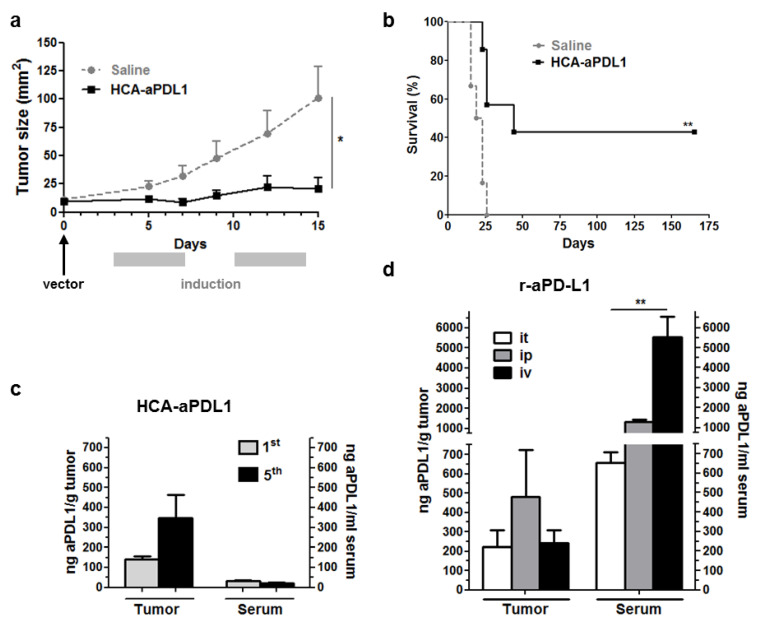
Intratumoral administration of HCA-EFZP-aPDL1 allows local expression of aPD-L1 and inhibition of subcutaneous tumor growth. Tumors were established by subcutaneous inoculation of MC38 cells. The vector (1 × 10^10^ vg in 50 µL) was injected intratumorally (day 0), and the expression of aPD-L1 was activated by i.p. administration of mifepristone at days 3–7 and 10–14. (**a**) The progression of subcutaneous tumors was evaluated in mice receiving the vector (HCA-aPDL1) or saline solution as a control group (*n* = 7), by direct measurement. * *p* < 0.05, Mann–Whitney U test. (**b**) Survival curve. ** *p* < 0.01 log-rank test. Data are represented as average ± SEM. (**c**) A subset of animals (*n* = 5) was sacrificed 10 h after the 1st and 5th mifepristone injections (days 4 and 8) for quantification of aPD-L1 in serum and tumor extracts. (**d**) For comparison of mAb biodistribution, additional groups of mice (*n* = 4) received the recombinant mAb (100 μg every 72 h for 3 doses) following the intratumoral (it), intraperitoneal (ip) or intravenous (iv) routes. Mice were sacrificed 8 h after the last administration for quantification of aPD-L1. ** *p* < 0.01 Kruskal–Wallis test.

Next, subcutaneous tumors were established by implantation of MC38 cells, a preclinical model of microsatellite-instable colorectal cancer [20]. The HCA-EFZP-aPD-L1 vector (1 × 10^10^ vg/mouse in 50 µL) was injected locally, and transgene expression was activated by mifepristone following the same regime indicated above. The follow-up of mice revealed a significant inhibition of tumor growth and increased survival (Figure 2a,b, respectively), indicating the safety and potential efficacy of this approach. When the same dose of vector was administered in the absence of mifepristone induction, the antitumor effect was marginal (Supplementary Figure S1). These results confirm that the HCA-EFZP-aPD-L1 vector achieves local and controlled expression of aPD-L1. A subset of mice was sacrificed 10 h after the first or fifth mifepristone induction in order to confirm the presence of aPD-L1 in the tumor microenvironment. As show in Figure 2c, the concentration reached more than 300 ng aPD-L1/g of the tumor, whereas very low levels of the mAb were detected in serum. In contrast, high doses of the mAb used as a recombinant protein (100 μg every 72 h for three doses) were needed to obtain similar intratumoral concentrations, following different routes of administration (intratumoral, i.p. or i.v.). In this case, the levels were higher in serum than in tumors, especially in the case of the i.v. route (Figure 2d). Of note, the regime based on i.p. injections has shown a partial therapeutic effect in this tumor model [15], consistent with the access of the mAb to the tumor. 

**Figure 3:**
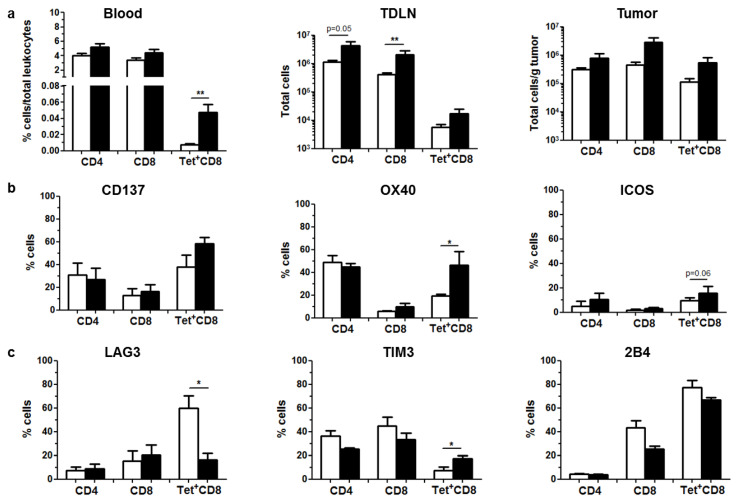
Local expression of aPD-L1 stimulates antitumor immune responses. C57BL/6 mice bearing subcutaneous MC38 tumors (*n* = 5) were treated as described in Figure 2. Mice were sacrificed after the 5th day of aPD-L1 expression for collection of blood, TDLNs and tumors. Immune cell populations were analyzed by flow cytometry. Control and HCA-EFZP-aPD-L1-treated groups are represented as white and black columns, respectively. (**a**) Quantification of CD4^+^, CD8^+^ and tumor-specific (Tet+) CD8^+^ T cells in peripheral blood (percentage of total leukocytes), TDLNs (total cells) and tumor extracts (total cells/g of tumor). (**b**) Percentage of cells expressing the stimulatory receptors CD137, OX40 and ICOS in the surface of these cell populations isolated from tumors. (**c**) Percentage of cells expressing the inhibitory receptors LAG3, TIM-3 and 2B4 in the same cells. * *p* < 0.05, ** *p* < 0.01, Mann–Whitney U test. Data are represented as average ± SEM.

A subset of control and vector-treated mice was sacrificed 5 days after the activation of aPD-L1 expression for collection of blood, tumor-draining lymph nodes (TDLNs) and tumors. In line with previous results using other vectors for expression of aPD-L1 [15], the analysis of antitumor immune responses revealed an overall stimulation in treated mice (Figure 3). An increase in CD4 and CD8 T lymphocytes was detected in the tumor microenvironment and the draining lymph nodes of treated mice. In addition, CD8^+^ T lymphocytes specific for the KSPWFTTL tumor epitope identified by tetramer staining were significantly elevated in peripheral blood (Figure 3a). The presence of some activating receptors such as OX40 and ICOS was increased in the surface of CD8 cells in the tumor (Figure 3b), whereas the inhibitory receptor LAG3 was reduced in tumor-specific CD8^+^ T lymphocytes. In addition, the activation of immune-modulatory mechanisms was indicated by elevation of TIM-3 in this cell population (Figure 3c).

### 2.2. Colorectal Cancer Peritoneal Metastases Are Refractory to aPD-L1 Treatment

**Figure 4:**
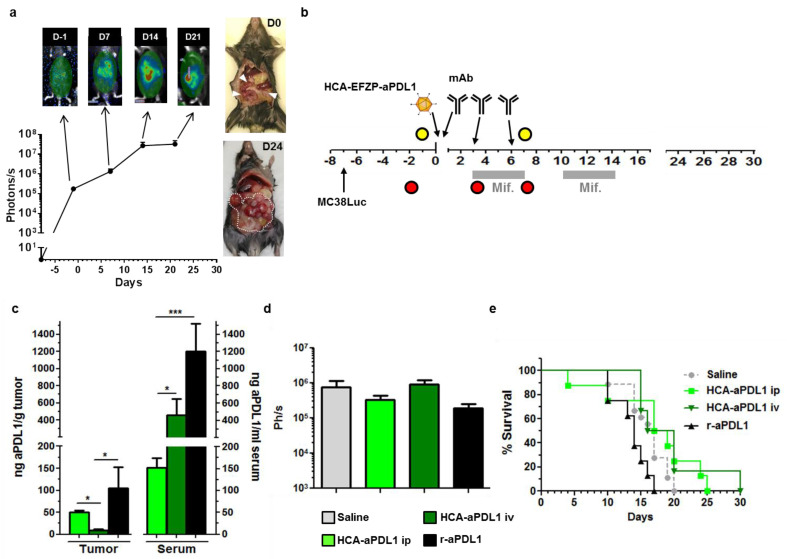
Colorectal cancer peritoneal metastases are refractory to aPD-L1 treatment. (**a**) Characterization of the PC model. I.p. injection of MC38Luc1 cells leads to rapid progression of tumors. Light emission from tumors can be detected by BLI one week after cell implantation (considered as day-1, D-1) and shows a progressive increase during the following 2 weeks. The graph represents quantification (in photons/s) from the abdominal area of mice at different time points (*n* = 12 days 0–14, *n* = 4 day 21). Images on top correspond to representative mice during BLI. The pictures on the right show tumor extension at days 0 and 24. (**b**) Schematic representation of the treatment schedule. The HCA-EFZP-aPD-L1 vector (HCA-aPDL1) was administered at 10^11^ vg/mouse by i.p. or i.v. injection (*n* = 10 and *n* = 6, respectively) at day 0 (one week after cell inoculation). Mifepristone induction consisted of 2 cycles during days 3–7 and 10–14. The recombinant aPD-L1 mAb (r-aPDL1) was administered by i.p. injection (*n* = 6) on days 0, 3 and 6. Yellow and red circles indicate BLI and blood collections, respectively. (**c**) Additional groups of mice (*n* = 6) received the HCA-EFZP-aPD-L1 vector by i.v. or i.p. injection, or the recombinant protein (*n* = 5). Mice were sacrificed 10 h after the first mifepristone induction, or 8 h after the last protein administration for quantification of aPD-L1 concentration in serum and tumor extracts. (**d**) Light emission from control and treated mice at day 7. (**e**) Survival curves. * *p* < 0.05, *** *p* < 0.001, Mann–Whitney U test. Data are represented as average ± SEM.

In order to check the efficacy of HCA-EFZP-aPD-L1 in more stringent, clinically relevant settings, first, we evaluated the antitumor effect of the treatment in larger subcutaneous tumors. We followed the same protocol described above, but the vector was administered 16 days after cell implantation, when the average tumor size was 43.5 ± 6.2 mm^2^. Under these experimental conditions, only a small, non-significant delay of tumor growth was observed (Supplementary Figure S2). Next, we employed a model of peritoneal carcinomatosis (PC) based on the i.p. injection of MC38Luc1 cells. This is a very aggressive model, with rapid progression of tumors and a median survival of 3 weeks after cell implantation in untreated mice. Expression of PD-L1 was confirmed in tumor cells and the leukocyte infiltrate by flow cytometry (Supplementary Figure S3). Stable expression of luciferase in the cancer cells allows non-invasive longitudinal assessment of tumor growth and dissemination by bioluminescence imaging (BLI). Light emission can be detected one week after injection of the tumor cell suspension, coinciding with the appearance of macroscopic tumors attached to the peritoneum (considered as day-1 in Figure 4a). The signal increases during the next 2 weeks, followed by a plateau in which it is not fully representative of the tumor burden. This phenomenon has been previously described in a model of liver metastases [21] and is mainly dependent on changes in the composition of the tumor tissue (cancer vs. stromal cells) and the appearance of physical barriers for light penetration such as hemorrhagic ascites. Although PC is an advanced stage of colorectal cancer, it is amenable to regional administration, with the aim of reducing the systemic toxicity of the therapeutic agents. Therefore, a relatively high dose of the HCA-EFZP-aPD-L1 vector (10^11^ vg in 100 μL) was administered by i.p. injection one week after cell inoculation. For comparison, the same dose of vector was administered to a different group of mice by i.v. administration. Finally, an additional group received aPD-L1 as a recombinant protein by i.p. injection, following a standard regime of 100 μg on days 0, 3 and 6 (Figure 4b). The time of vector injection is considered as day 0 in the scheme provided in Figure 4b. Mifepristone induction was initiated two days later and consisted of two cycles of 5 consecutive days with a 2-day resting period between them. The dose of mifepristone was 1 and 4 mg/Kg for the first and second induction cycles, respectively. Luciferase activity was quantified before vector administration to ensure cancer cell engraftment, and one day after completion of the first cycle (day 7 in Figure 4b). Blood was collected in the basal state (day-2), and 10 h after the first induction (day 3). A subset of control and vector-treated mice was sacrificed at this point in order to determine the concentration of aPD-L1 in serum and tumor extracts. In the case of mice receiving the recombinant mAb, samples were collected 8 h after the last administration. Evaluation of the antitumor effect was performed by BLI at day 7 and by comparison of survival curves. The concentration of aPD-L1 was close to background before mifepristone induction (not shown). As shown in Figure 4c, the i.p. route of the vector obtained a more favorable ratio between intratumoral and systemic concentrations of the antibody, in comparison with the i.v. route. The i.p. injection allowed access of the vector and expression of aPD-L1 into disseminated tumor foci, although the limitation of aPD-L1 release into the circulation was only partial in comparison with the intratumoral route described in Figure 2c. As previously observed in the subcutaneous tumors, the recombinant protein was detected in the peritoneal implants, but the levels in blood were much higher than those found after i.p. administration of the vector. Despite the preferential location of anti-PD-L1 in the tumor mass, we could demonstrate neither a reduction in the luciferase signal nor a significant improvement in survival in vector-treated mice (Figure 4d,e). The same outcome was observed in animals receiving the vector by i.v. administration, despite the high levels of antibody observed in serum. In the case of the recombinant antibody, a small, non-significant reduction in the luciferase signal was observed at day 7, but no improvement in survival could be demonstrated. These results indicate that, although the tumors are not intrinsically resistant to aPD-L1, the progression of peritoneal metastases cannot be controlled by this treatment in any of the modalities tested. Persistence of the vector was demonstrated in liver and tumor samples obtained in mice treated with the same dose of vector and sacrificed at days 3, 7 and 10. As expected, vector genomes were more stable in the liver than in tumors (Supplementary Figure S4). 

### 2.3. Lack of Synergy between HCA-EFZP-aPD-L1 and Other Immune-Stimulatory Agents in the PC Model

**Figure 5:**
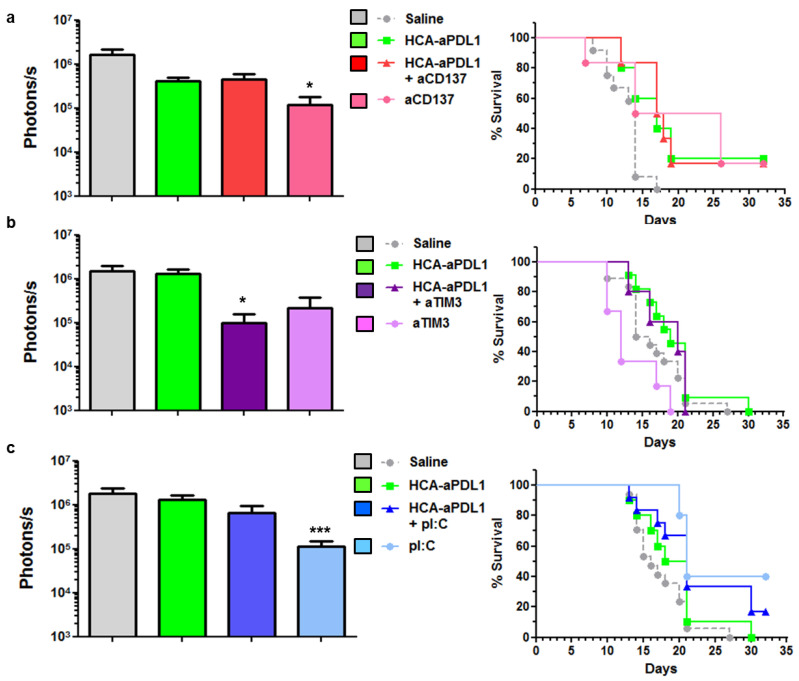
Combination of HCA-EFZP-aPDL1 and aCD137, aTIM-3 or pI:C in the PC model. Tumors were established and treated with HCA-EFZP-aPDL1 as described in Figure 4. (**a**) The aCD137 mAb was administered by i.p. injection on days 0 and 3 at 200 µg per dose, alone or in combination with the vector (*n* = 6). (**b**) The aTIM-3 mAb was administered on days 2 and 5 at 300 µg per dose (*n* = 6). (**c**) pI:C was administered on days 3 and 10 at 50 µg per dose (*n* = 10). The control group (*n* = 12) received an i.p. injection of saline solution. The left panels show light emission from tumors at day 7, and right panels are survival curves. * *p* < 0.05, *** *p* < 0.001, Kruskal–Wallis test (photons/s) or log-rank test (survival). Data are represented as average ± SEM.

We employed the PC model for the search of methods to enhance the efficacy of aPD-L1. To this end, the i.p. administration of HCA-EFZP-aPDL1 was combined with different immune-stimulatory agents. Among all possibilities, the candidates were chosen based on the analysis of the tumor microenvironment (Figure 3) and our previous experience with local expression of aPD-L1 using an SFV vector [15]. In all cases, the induction regime with mifepristone was the same as described in Figure 4b. First, we tested the agonistic anti-CD137 antibody, since it can enhance the antitumor effect of SFV-aPDL1 in the subcutaneous MC38 model. Following the same treatment schedule described in Figure 4b, the aCD137 mAb was administered i.p. on days 0 and 3, at 200 μg per dose, alone or in combination with the HCA-EFZP-aPD-L1 vector. Quantification of the luciferase signal at day 7 after vector administration (Figure 5a, left panel) revealed a moderate but significant decrease in mice receiving aCD137 as monotherapy (control: 1.66 × 10^6^ ± 4.82 × 10^4^, *n* = 12; aCD137: 1.2 × 10^5^ ± 6 × 10^4^ photons/s, *n* = 6, *p* = 0.008 Kruskal–Wallis test). This effect was corroborated by a slight increase in survival in the aCD137 group (Figure 5a, right panel). However, the combination with HCA-EFZP-aPDL1 did not increase the therapeutic effect. 

Next, we tested the combination of the vector with the blocking mAb against TIM-3, since this target is highly expressed in cancer-specific CD8^+^ T cells found in tumors treated by HCA-EFZP-aPDL1 (Figure 3c). The mAb was administered i.p. on days 2 and 5 at 300 μg per dose. Although a partial reduction in tumor progression was observed in the BLI assays in mice treated with the combination (control: 1.47 × 10^6^ ± 4.58 × 10^5^, *n* = 12; combination: 9.92 × 10^4^ ± 6 × 10^4^ photons/s, *n* = 5, *p* = 0.008, Kruskal–Wallis test), no improvement in survival was observed (Figure 5b). Finally, the HCA-EFZP-aPDL1 vector was combined with polyinosinic-polycytidylic acid (pI:C), in order to stimulate type I interferon responses in the tumors. This is a key component in the antitumor effect of SFV-aPDL1 [15], and it has shown a strong therapeutic effect in combination with recombinant aPD-L1 in a subcutaneous MC38 tumor model [22]. pI:C was administered by i.p. injection on days 3 and 10 at 50 µg per dose resuspended in 100 µL saline solution. As observed with the immune-stimulatory aCD137 mAb, pI:C alone was able to reduce tumor progression (control: 1.81 × 10^6^ ± 4.93 × 10^5^, *n* = 17; pI:C: 1.14 × 10^5^ ± 3.73 × 10^4^, *n* = 10, *p* = 0.0005, Kruskal–Wallis test). As for survival, all groups receiving pI:C showed a significant increase compared with untreated animals (*p* = 0.01 and *p* = 0.009 log-rank test for pI:C and combination, respectively). However, no cooperation between pI:C and HCA-EFZP-aPDL1 was observed (Figure 5c). 

### 2.4. Depletion of Macrophages Increases the Survival of Mice Treated with HCA-EFZP-aPDL1

**Figure 6:**
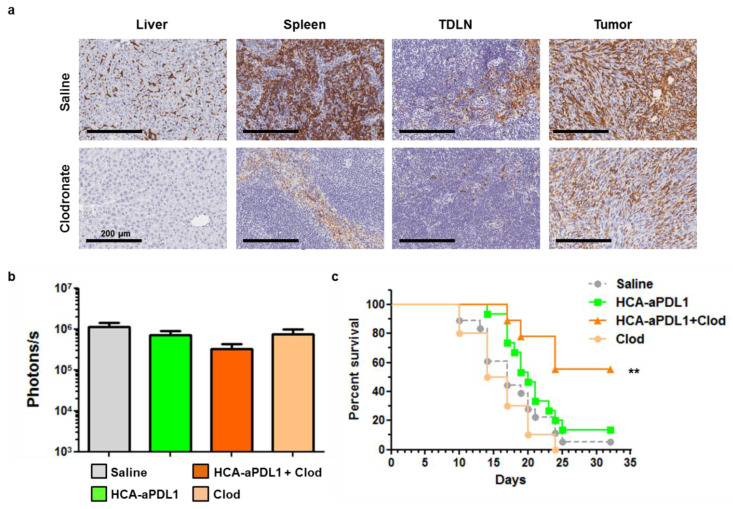
Depletion of macrophages increases the survival of mice treated with HCA-EFZP-aPDL1 in the PC model. Tumors were established and treated with HCA-EFZP-aPDL1 as described in Figure 4. Clodronate-loaded liposomes (0.5 mg clodronate in 100 µL) were administered i.v. on days 1 and 8, alone or in combination with the vector (*n* = 9). (**a**) A subset of mice was sacrificed 24 h after clodronate treatment for identification of macrophages in the indicated tissues by immunohistochemistry. (**b**) Light emission from tumors at day 7. (**c**) Survival curve. ** *p* < 0.01, log-rank test. Data are represented as average ± SEM.

After the lack of cooperation between HCA-EFZP-aPDL1 and the treatments described above in the PC model, we targeted other potential sources of tumor resistance such as macrophages. The MC38 tumor model is characterized by a diverse composition of the leukocyte infiltrate, with a progressive expansion of macrophages and a concomitant reduction in their pro-inflammatory (M1) phenotype [23]. They present reduced IL-12 and iNOS signaling, and activation of the IL-10 pathway [24]. In order to reduce the influence of macrophages in the TME, tumor-bearing mice received i.v. injections of clodronate-loaded liposomes (100 µL at 5 mg/mL) on days 1 and 8, alone or in combination with the vector. A subset of mice treated with the clodronate formulation was sacrificed 24 h later to confirm depletion of macrophages. As observed in Figure 6a, this cell population was efficiently eliminated from the liver (Kupffer cells) and TDLNs (inguinal lymph nodes in this case) and partially from the spleen and tumors. 

The analysis of tumor progression by BLI showed only a moderate reduction in luciferase activity in the combination group (*p* = 0.13, Figure 6b). In contrast, this group showed a robust increase in survival (*p* = 0.003 control vs. combination, *p* = 0.02 HCA-EFZP-aPDL1 vs. combination, log-rank test), with 55% mice alive one month after the initiation of treatment (Figure 6c). Interestingly, just the depletion of macrophages showed no antitumor effect.

## 3. Discussion

Local expression of aPD-L1 using gene therapy vectors has been recently proposed to improve the pharmacokinetics of this mAb [15]. In this approach, the properties of vectors have shown a great influence in the response to the treatment. The intrinsic immunostimulatory properties of SFV were instrumental for its antitumor effect, in contrast with AAV. In this work, we have demonstrated that HC-AdVs can be employed to achieve controlled and sustained expression of aPD-L1, resulting in a significant antitumor effect in a subcutaneous colorectal cancer model. However, the efficacy of ICIs targeting the PD-1/PD-L1 axis depends not only on the intrinsic characteristics of tumor cells but also on compensatory mechanisms and on the composition of the TME [3,12]. We show here that the same type of tumor becomes largely insensitive to aPD-L1 expression when it grows as a rapidly progressing PC, in contrast with the subcutaneous nodules. Our results indicate that the limiting factor is not the amount of aPD-L1 present in the circulation, since the recombinant mAb administered as a high-dose i.p. bolus or the i.v. administration of HCA-EFZP-aPDL1 was inefficacious as well. In the latter case, high levels of aPD-L1 are produced and secreted from the liver into the bloodstream. These results are in line with the limited clinical benefit observed in advanced colorectal cancer patients treated with PD-1/PD-L1 modulators [25]. The intratumoral concentration of the antibody may be one limiting factor, together with the development of secondary resistances. In our search for potential solutions, we found that other immune-stimulating agents such as aCD137, aTIM-3 or pI:C exert partial antitumor effects in this stringent cancer model, but they do not synergize with aPD-L1 expression. In contrast, a clear enhancement of efficacy was observed when HCA-EFZP-aPDL1 was used in combination with macrophage depletion. Tumor-associated macrophages (TAMs) with an M2 phenotype have been associated not only with failure of anti PD-1/PD-L1 therapy (reviewed in [26]) but also with hyper-progressions in lung cancer patients. The proposed mechanism relies on the activation of a subset of macrophages (epithelioid CD163^+^ CD33^+^ PD-L1^+^) through Fc/FcR interaction, inducing a marked immunosuppressive phenotype in these cells [27]. M2 macrophages tend to express more PD-L1 than M1 macrophages, and they accumulate in the TME of advanced cancers [26,28]. Administration of clodronate-loaded liposomes is an efficient and affordable way to deplete macrophages in several tissues of mice (including tumors [29]), and it has been instrumental to demonstrate the immunosuppressive role of these cells. In our experimental setting, depletion was very efficient in the liver, followed by TDLNs and the spleen, and only partially efficient in tumors. We hypothesize that depletion in TDLNs plays a crucial role in the enhancement of anti-PD-L1 effects observed in the PC model. Until clodronate is approved for systemic administration, other clinically compatible methods could achieve the same results. Intravenous administration of gadolinium chloride can block Kupffer cells [30], although its efficacy in extra-hepatic tissues is uncertain. Another option is to inhibit macrophage recruitment in tumors, by blockade of the chemokines colony stimulating factor 1 (CSF-1), C-C motif chemokine ligand 2 (CCL2) or their receptors [31,32]. Importantly, cooperation between anti-PD-L1 therapy and the CSF-1R inhibitor PLX3397 has been recently described in a mouse model of hepatocellular carcinoma [32]. In addition, M2 polarization could be avoided by treatment with bis-phosphonates such as zoledronate [33,34], the chemotherapeutic agent trabectedin [35], the Hedgehog inhibitor Vismodegib [36] or new PI3Kγ inhibitors [24]. Further evaluation of these agents in combination with aPD-1/PD-L1 therapy is warranted. 

In conclusion, we have developed a new gene therapy tool for sustained and controlled expression of the aPD-L1 mAb. Using a stringent PC model, we have identified macrophages as one important target to avoid resistance to aPD-L1. This information can guide future clinical investigations in advanced colorectal cancer and other malignancies.

## 4. Materials and Methods 

### 4.1. Plasmids and Vector Construction

The sequence encoding a mAb against mouse PD-L1 has been previously described [15]. In this construct, the heavy and light chains of the chimeric hamster/mouse IgG2a are linked by a foot and mouse disease virus 2A autoprotease sequence preceded by a furin protease cleavage site (Figure 1a). For the construction of the adenoviral genome, the aPD-L1 sequence was excised from the pAAV-aPDL1m plasmid using EcoRV restriction sites and introduced into the pAsc-EFZP plasmid [37] containing the ubiquitous mifepristone-inducible system. The complete expression cassette was then introduced into the pD23-E4 plasmid using AscI restriction sites. pD23-E4 is a derivative of the pDelta28-E4 plasmid (courtesy of Brendan Lee, Baylor College of Medicine, Houston, TX, USA [38]). It contains the left ITR and packaging signal from human adenovirus type 5 followed by stuffer DNA from human origin, the E4 promoter region and the right ITR. The resulting pD23-EFZP-aPDL1 plasmid was linearized by PmeI digestion and transfected into 293Cre4 cells. Particle rescue and amplification was performed by concomitant infection with the AdTetCre helper virus, as previously described [39]. Viral particles were released from cells by three cycles of freezing and thawing. After the last amplification step, the vector was purified by double ultracentrifugation in cesium chloride gradients and desalted using a Sephadex G-50 column (Sigma, St. Louis, MO, USA). They were formulated in Tris pH 8.1 with 10% glycerol and stored at −80 °C. Titration of the purified vectors was carried out by PCR quantification of Hirt-extracted genomes [40]. Titers are expressed as viral genomes (vg). 

### 4.2. Cell Culture

MC38Luc1 cells were obtained by stable transfection of the pCMV-Luc plasmid in the mouse colorectal cancer cell line MC38, as previously described [21]. Cells were maintained in Dulbecco’s modified Eagle’s medium supplemented with 10% heat-inactivated fetal bovine serum, 100 mg/mL streptomycin and 100 mg/mL penicillin (all from Gibco, Invitrogen, Paisley, UK) and 2 mM glutamine (Cambrex, Wiesbaden, Germany). In the case of MC38Luc1, the media were supplemented with 400 mg/mL G418 (Geneticin, Gibco, Invitrogen, Paisley, UK) and removed 48 h before inoculation in mice.

### 4.3. Animal Experimentation Procedures and Reagents

All in vivo studies were performed in female C57BL/6 mice (Envigo, Barcelona, Spain) following protocols approved by the Ethic Committee for Animal Experimentation from the University of Navarra (CEEA). Protocol codes are: 099-14 (2 October 2014), 024-18 (12 April 2018) and 086-16 (27 July 2016). For the subcutaneous tumor model, 5 × 10^5^ MC38 cells resuspended in 100 µL of saline solution were inoculated into the right hind flank of mice. When tumor nodules reached at least 3.5 mm in diameter (typically 7–9 days after cell inoculation), the vector was administered intratumorally, resuspended in 50 µL of saline solution. Induction of aPD-L1 expression was performed by i.p. administration of mifepristone (RU486, Sigma, St. Louis, MO, USA) dissolved in 100 μL of sesame oil (Sigma, St. Louis, MO, USA), using the schedules indicated in the Results section. Tumor progression was evaluated by direct size measurement using a caliper. Values are represented as mm^2^, calculated based on the area of an ellipse using the following formula: *R* × *r* × π, where *R* and *r* are the major and the minor radius, respectively. Blood collection was performed by maxillary vein puncture. Animals were euthanized if their general status deteriorated or subcutaneous tumors exceeded 20 mm in diameter. 

For the PC model, 5 × 10^5^ MC38Luc1 cells resuspended in 100 µL of saline solution were inoculated i.p. BLI was used for non-invasive estimation of tumor progression. To this end, mice were anesthetized with i.p. injection of a ketamine/xylacine mixture. The substrate D-luciferin (REGIS Technologies, Chicago, IL, USA) was administered i.p. (100 μL of a 30 μg/μL solution in PBS) and light emission was detected using a PhotonImager Optima apparatus (BioSpace, Paris, France) at different times post-luciferin injection (5, 10, 15 and 30 min). The highest value (photons/s) corresponding to a fixed region of interest (ROI) encompassing the abdomen was selected from each animal. Approximately one week after cell implantation, the first BLI determination was performed to guarantee the presence of tumors in all animals before initiation of treatment. The vector was administered by i.p. or i.v. injection in a volume of 100 µL of saline solution. The mouse anti-CD137 (clone 3H3) and anti-TIM-3 antibodies (clone RMT3-23) were from BioXcell. Mice received 200 μg (anti-CD137) or 300 μg (anti-TIM-3) by i.p. injection, following the schedule indicated in the Results section. pI:C (Sigma, St. Louis, MO, USA) was administered i.p. at 50 μg. Clodronate liposomes (Liposoma BV, Amsterdam, NL, USA) were administered i.v. (0.5 mg in 100 μL). Animals were euthanized if their general status deteriorated (reduced mobility, unable to feed) according to the approved endpoints. 

### 4.4. Quantification of Anti-PD-L1

Development of the PD-L1-binding ELISA was previously described [15]. Briefly, plates were first coated with 1 μg/mL recombinant murine PD-L1 fused to human IgG1 Fc (R&D, Minneapolis, MN, USA) and incubated overnight at 4 °C. Then, serial dilutions of samples (serum or tumor lysates) were added. Plates were then incubated with a goat polyclonal secondary antibody anti-mouse IgG2a conjugated with peroxidase (Abcam, Cambridge, UK), and the assay was developed with tetramethylbenzidine (TMB) substrate. The absorbance was measured in an ELISA reader at 450 nm. Serum was obtained by centrifugation of blood at 10,600× *g* for 10 min. Tumor samples were homogenized in 250 mL of PBS-Tween 0.05% in the presence of protease inhibitor cocktail tablets (Roche, Mannheim, Germany) and centrifuged at 10,600× *g* for 10 min to obtain the tissue extracts. In the case of peritoneal tumors, intracardiac perfusion with PBS was performed before tissue collection in order to wash out blood from tissues. To this end, PBS was injected in the left ventricle of anesthetized mice at a constant rate of 6.5 mL/min for 2 min using a perfusion pump. The excess fluid was eliminated through an incision in the right atrium.

### 4.5. Flow Cytometry Analysis 

Tumors and tumor-draining lymph nodes (TDLNs) were treated with 400 U/mL collagenase D and 50 mg/mL DNase I (Roche Diagnostics, Indianapolis, IN, USA). After mechanical tissue dissociation, cells were passed through a 70-mm nylon mesh filter (BD Falcon, BD Biosciences, San Jose, CA, USA), washed, treated with ACK lysing buffer and washed again. Blood samples were surface stained and then fixed, and erythrocytes were cleared with FACS Lysing Solution (BD Biosciences, San Jose, CA, USA). In all cases, a single-cell suspension was pretreated with anti-CD16/32 (clone 2.4G2; BD Pharmingen, San Jose, CA, USA) to reduce non-specific binding to Fc receptors. After this, cells were stained with the following fluorochrome-conjugated antibodies: CD8 (clone 53-6.7), CD4 (clone RM4-5), LAG-3 (clone C9B7W), CD137 (clone 17B51H1), OX40 (clone OX-86), TIM-3 (clone RMT3-23), ICOS (clone C398.A4) and 2B4 (clone m2B4 (B6)458.1), all of them from Biolegend (San Diego, CA, USA). To identify tumor-specific CD8 T lymphocytes, we stained cells with H-2Kb MuLV p15E Tetramer-KSPWFTTL (MBL International, Woburn, MA, USA), which includes a peptide (p15E6_04-611_) from the envelope protein of an endogenous ecotropic murine leukemia virus [41]. This immunodominant epitope is presented by different tumors originated from C57BL/6 mice, such as MC38 and B16 [42]. PD-L1 expression in tumor cells and leukocyte infiltrate was performed as previously described [15]. A FACS Canto II (BD Biosciences, Franklin Lakes, NJ, USA) was used for cell acquisition, and data analysis was carried out using FlowJo software (Tree Star, Ashland, OR, USA).

### 4.6. Immunohistochemistry

Tissue samples were fixed in 4% formaldehyde (Panreac, Barcelona, Spain) for 24 h and then incubated in 70% ethanol for an additional 24 h before paraffin embedding. Serial paraffin sections (3 μm thick) were cut and treated with Proteinase K (Roche, Mannheim, Germany). Primary antibody (rat F4/80, BioLegend, San Diego, CA, USA) was used at 1:400 dilution. Detection was performed by Rabbit anti-rat E0468 (Dako-Agilent, Santa Clara, CA, USA).

### 4.7. Statistical Analysis

Data were processed for statistical analysis using the Graphpad Prism software (San Diego, CA, USA) Normality was assessed with the D’Agostino and Pearson omnibus normality test. Multiple groups were compared using 1-way ANOVA with Tukey’s post-test, or Kruskal–Wallis with Dunn’s post-test, for normal and non-normal distributions of data, respectively. Comparison of two groups was performed by the Mann–Whitney U test. Survival curves were compared using the log-rank test. The significance level was set at *p* < 0.05.

## Data Availability

Not applicable.

## Supplementary Materials

The following are available online at https://www.mdpi.com/article/10.3390/ijms22084176/s1, Supplementary Figure S1. Intratumoral injection of HCA-EFZP-aPDL1 has no therapeutic effect in the absence of mifepristone administration. Supplementary Figure S2. Intratumoral injection of HCA-EFZP-aPDL1 has no therapeutic effect if the treatment is delayed. Figure S3. Cancer and stromal cells express PD-L1 in MC38 peritoneal tumors. Supplementary Figure S4. Persistence of vector in tumor and liver tissue in the PC model.
