# Supplementary material for: Adenovirus-Mediated Inducible Expression of a PD-L1 Blocking Antibody in Combination with Macrophage Depletion Improves Survival in a Mouse Model of Peritoneal Carcinomatosis
Source: Int J Mol Sci. 2021 Apr 17;22(8):4176. doi: 10.3390/ijms22084176 (PMC8073765; doi:10.3390/ijms22084176)
Supplement: Supplementary file 1 [file ijms-22-04176-s001.zip › ijms-1092323-supplementary.pdf]

Supplemental Information

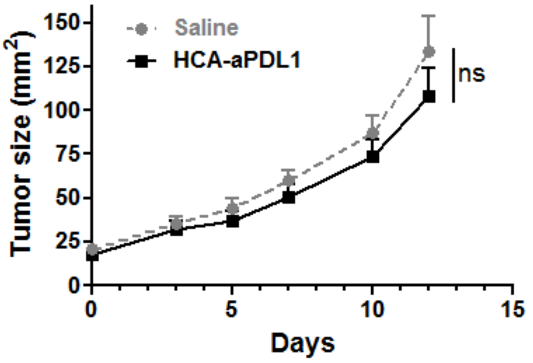

Figure S1. Intratumoral injection of HCA-EFZP-aPDL1 has no therapeutic effect in the absence of mifepristone administration. Tumors were established by subcutaneous inoculation of MC38 cells. The vector ($1x10^{10}$ vg in 50 μL) was injected intratumorally (day 0), but induction of transgene expression with mifepristone was not performed. The progression of subcutaneous tumors was evaluated in mice receiving the vector (HCA-aPDL1) or saline solution as a control group (n=6), by direct measurement. ns, non-significant.

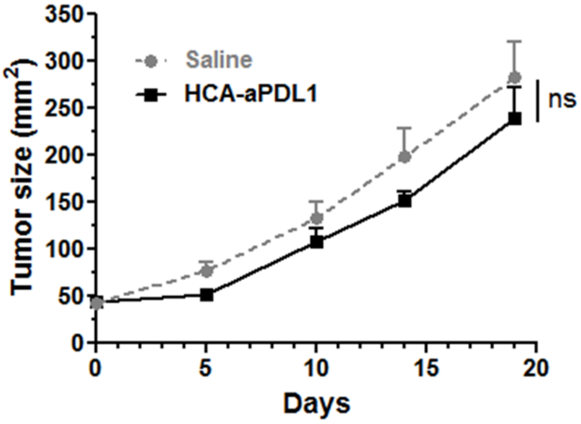

Figure S2. Intratumoral injection of HCA-EFZP-aPDL1 has no therapeutic effect if the treatment is delayed. Tumors were established by subcutaneous inoculation of MC38 cells. The vector ($1x10^{10}$ vg in 50 μL) was injected intratumorally 16 days after cell implantation (day 0), when tumors reached an average size of 43.5 ±6.2 mm^2. The expression of aPD-L1 was activated by i.p. administration of mifepristone at days 3-7 and 10-14. The progression of subcutaneous tumors was evaluated in mice receiving the vector (HCA-aPDL1) or saline solution as a control group (n=5), by direct measurement. ns, non-significant.

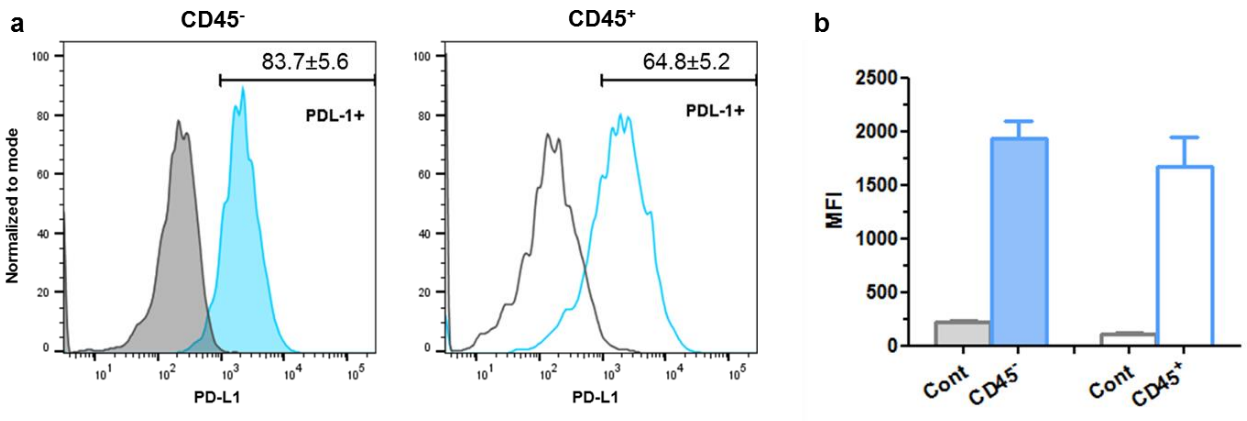

Figure S3. Cancer and stromal cells express PD-L1 in MC38 peritoneal tumors. Peritoneal tumors were obtained by injection of $5x10^5$ MC38Luc1 cells in the peritoneum of C57BL/6 mice. Sixteen days later, mice were sacrificed and cells were isolated from tumors for analysis of PD-L1 expression by flow cytometry. **a**, representative histograms of cancer cells ($CD45^-$, filled) and leukocyte infiltrate ($CD45^+$, empty), indicating the percentage of cells expressing PD-L1. **b**., mean fluorescence intensity (MFI). In all cases the gray histograms or bars represent isotype controls.

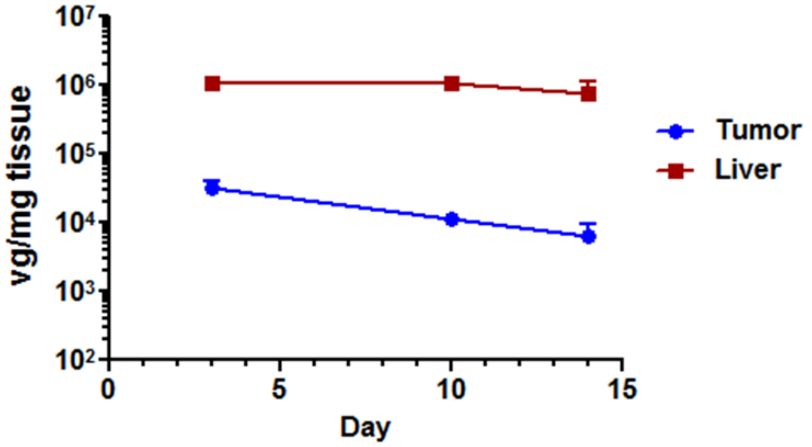

Figure S4. Persistence of vector in tumor and liver tissue in the PC model. Tumors were established by i.p. injection of MC38Luc1 cells. The HCA-EFZP-aPD-L1 vector was administered at 10^{11} vg/mouse by i.p. injection at day 0 (one week after cell inoculation). Mifepristone induction consisted of 2 cycles during days 3-7 and 10-14. Subgroups of mice (n=4) were sacrificed at the indicated times for liver and tumor collection. Viral genome copies (vg) were quantified by qPCR and expressed as vg/mg of tissue.
